# Emergent Virus Reactivation in SARS-CoV-2-Negative Community Acquired Pneumonia Patients During the COVID-19 Pandemic

**DOI:** 10.3389/fmicb.2022.758073

**Published:** 2022-02-07

**Authors:** Junyan Qu, Fang He, Huan Li, Xiaoju Lv

**Affiliations:** Center of Infectious Disease, West China Hospital, Sichuan University, Chengdu, China

**Keywords:** community acquired pneumonia, herpesviridae, reactivation, clinical characteristics, pandemic

## Abstract

Emergent viruses (namely, HSV-1, CMV, and EBV) reactivation were common in critically ill patients and/or immunosuppressed patients. This study aimed to understand the clinical manifestations and reactivation of the emergent viruses in SARS-CoV-2-Negative community acquired pneumonia (CAP) patients during the COVID-19 pandemic. We retrospectively reviewed the medical records of CAP patients from January to March 2020, in our university hospital in China. The patients were divided into two groups based on the presence or absence of emergent viruses. In all patients, the positive rates of EBV, HSV, and CMV were 23.43% (15/64), 22.06% (15/68), and 12.50% (8/64), respectively. The most common presenting symptoms were fever (98, 57.99%) and dry cough (55, 32.54%). The levels of albumin, hemoglobin, lymphocyte count, and CD4 + T lymphocyte count in emergent viruses positive group were lower than those of viruses negative group (*P* < 0.05). The initial chest CT features of these patients were diverse. The most common manifestations were ground-glass opacity (91/169, 53.85%) and pulmonary nodule (88/169, 52.07%). More emergent viruses positive patients have bilateral upper lobes involvement than emergent viruses negative patients (*P* < 0.05). A total of 80.47% patients (136/169) received empirical antimicrobial treatment. The most commonly used antibiotic regimen was fluoroquinolone monotherapy (80/169, 47.34%). The emergent viruses positive patients have poorer clinical outcome (*P* < 0.05). In conclusion, emergent viruses reactivation was common in SARS-CoV-2-Negative CAP patients. Emergent viruses positive patients have poorer cellular immune function, more severer conditions and poorer prognosis. Fluoroquinolones may be a therapeutic option for CAP patients.

## Introduction

Since coronavirus disease 2019 (COVID-19) cases were first reported in Wuhan, China in December 2019, a large number of COVID-19 cases have appeared rapidly around the world WHO (2020). With the COVID-19 epidemic, there is an unprecedented focus on the diagnosis and treatment of patients with acute respiratory infection. Community-acquired pneumonia (CAP) is a common infection in the world. The common pathogens of CAP include *Streptococcus pneumoniae*, *Mycoplasma pneumoniae*, *Haemophilus influenzae*, *Chlamydia pneumoniae*, *Staphylococcus aureus*, and respiratory viruses (influenza, adenovirus, respiratory syncytial virus, and parainfluenza) (Mandell, 2015; Cao et al., 2018). The distribution of CAP pathogens varies significantly in different countries and regions and has changed over time, especially with the development of molecular diagnostics and the COVID-19 epidemic (Mandell, 2015; Cao et al., 2018; Rider and Frazee, 2018; Soo et al., 2020).

Both immunosuppression and immunoparalysis induced by the initial pro-inflammatory response to physiological insult can lead to the reactivation of latent viruses (Hotchkiss et al., 2013; Cantan et al., 2019). The emergent viruses, namely, herpes simplex virus type 1 (HSV-1), Epstein-Barr virus (EBV), and cytomegalovirus (CMV), were the most frequent reactivated viruses (Cantan et al., 2019). Recent research showed that emergent viruses reactivation in blood and/or lung could occur in intensive care unit (ICU) patients (Cantan et al., 2019). Emergent virus reactivation were associated with a poor outcome (Cantan et al., 2019; Goh et al., 2020). Although there have been many studies on CAP during COVID-19 epidemic (Metlay and Waterer, 2020; Zhou et al., 2020), data on herpesviridae (mostly HSV, CMV, and EBV) reactivation in CAP patients is rare.

In order to better understand the clinical manifestations and reactivation of the emergent viruses in CAP patients during the COVID-19 epidemic, we compared the clinical presentations, chest computerized tomography (CT) findings, therapeutic strategies and clinical outcomes in emergent viruses positive and negative CAP patients who were admitted to our hospital from 22 January 2020 to 12 March 2020.

## Materials and Methods

### Patients

From January 2020 to March 2020, the patients with SARS-CoV-2-Negative CAP patients were retrospectively reviewed in West China Hospital, Sichuan University, China (a 4,300-bed academic tertiary care hospital). The diagnosis of SARS-CoV-2-negative CAP according to the related literatures as defined as follows (Prina et al., 2015; National Health Commission of the People’s Republic of China, the State Administration of Traditional Chinese Medicine, 2021): (1) onset in community; (2) relevant clinical manifestations of acute lower respiratory tract infection: fever and/or respiratory symptoms, signs of pneumonia, new pulmonary infiltrate on chest CT; (3) at least two consecutive negative respiratory SARS-CoV-2 nucleic acid (sampling time interval of more than 24 h). Exclusion criteria were age under 14 years, confirmed COVID-19 patients (positive SARS-CoV-2 nucleic acid by RT PCR in respiratory or blood specimens or highly homologous to SARS-CoV-2 by gene sequencing of a respiratory or blood specimens), individuals recovered from asymptomatic SARS-CoV-2 infection, tuberculosis, pulmonary tumor, non-infectious interstitial lung disease, pulmonary edema, pulmonary embolism, and pulmonary vasculitis.

The following data of demographic characteristics, underlying diseases, initial clinical presentations, laboratory data, chest CT findings, and clinical outcomes were abstracted from the medical records and verified by two authors independently. Patients were divided into two groups based on the presence or absence of emergent viruses.

### Screening Process

The following epidemiological history of all patients was collected: (1) travel history or residence history of Wuhan or other affected areas within 14 days before onset; (2) a history of contact with confirmed COVID-19 patients within 14 days before onset; (3) a history of contact with suspected COVID-19 patients from affected areas within 14 days before onset; and (4) clustering occurrence.

**FIGURE 1:**
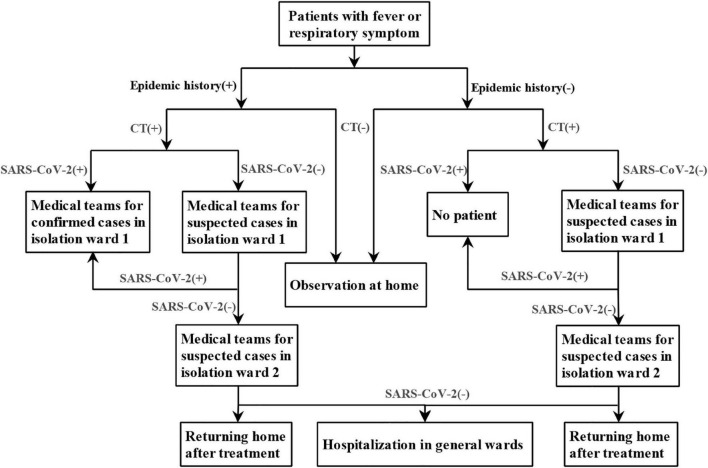
The screening flowchart of COVID-19 in West China Hospital, Sichuan University.

The screening flowchart of COVID-19 was shown in Figure 1, which was continuously improved and refined with deeper understanding of the disease. In order to reduce the chance of cross infection, active area of febrile patients, entrance of febrile patients, route of radiological examination for common febrile patient, and suspected or confirmed COVID-19 patients were set up separately. Different rooms for CT examinations were also set up for suspected and confirmed COVID-19 patients and other patients. Before and after each suspected patient underwent CT, the CT rooms were strictly sterilized.

### Laboratory Studies

Laboratory tests such as blood routine, blood biochemistry, procalcitonin (PCT), C-reactive protein (CRP) and interleukin-6 (IL-6), T lymphocyte subset, and serum immunoglobulin were performed. The nucleic acid of SARS-CoV-2 by reverse transcription-polymerase chain reaction (RT-PCR) in respiratory and stool specimens. Immunoglobulin M (IgM) and IgG antibodies to CMV and HSV in blood were measured by chemiluminescent immunoassay (CLIA) technology on the Diasorin LIAISON. The normal IgM antibody values of CMV and HSV are less than 22 U/mL and less than 1.1 Index, respectively. CMVDNA and EBVDNA levels in blood were assessed by quantitative PCR with the detection limit of 50 copies/mL. Throat swabs were screened by PCR for the nucleic acid of influenza virus (IFV) A, B, H1N1 and H3N2, human rhinovirus (HRV), adenovirus (AdV), human bocavirus (HBoV), respiratory syncytial virus (RSV), human metapneumovirus (hMPV), parainfluenza virus (PIV), human coronavirus, *Mycoplasma pneumoniae*, and *Chlamydia pneumoniae*. Human immunodeficiency virus (HIV) p24 antigen and HIV antibodies were detected by electrochemiluminescent immunoassay (ECLIA, Roche Diagnostics). The positive samples in the initial screening test were confirmed by western blot method and HIV nucleic acid. The lower limit of detection of HIVRNA was 20 copies/mL. Smear staining and culture of bacteria and fungi were performed when respiratory specimens available. Some patients were also screened for 1,3-β-D glucan test (G test), galactomannan (GM) antigen detection and interferon-gamma (IFN-γ) release assays (IGRAs) according to their clinical symptoms and imaging findings.

### Radiological Assessment

All suspected COVID-19 patients were scanned by 64-row multi-slice spiral CT (SOMATOM definition AS+, Siemens) in our hospital. Chest CT with consecutive 5-mm thick sections were obtained. Some patients were screened using high resolution CT scans with 1-mm collimation. All images were reviewed by two thoracic radiologists and clinicians (experts in infectious diseases, respiratory diseases, and severe diseases) who were unaware of the epidemiological history and clinical symptoms of the patients. Several radiological findings were recorded including ground-glass opacity (GGO), consolidation, interlobular septal thickening, patchy shadow, vascular enlargement, traction bronchiectasis, reticulation, and pleural thickening. The imaging features were defined as previous described (Ajlan et al., 2014). The lesion distribution in the lungs were also collected.

### Antimicrobial Treatment Strategies and Clinical Outcomes

Antibacterial, antiviral, and antifungal therapy were given according to the patients’ clinical diagnosis. Monotherapy was defined as use of one antibiotic or antiviral or antifungal drugs. Combination therapy was defined as the combination of antibiotics and antiviral drugs and/or antifungal drugs. Treatment outcome was evaluated at 28 days after the treatment. The continuous improvement of clinical symptoms and imaging findings were classified as improvement. Cure was defined as resolution of clinical symptoms and imaging findings.

### Statistical Analysis

Statistical analyses were performed with SPSS version 22.0 for windows (SPSS Inc., Chicago, United States). Continuous variables were presented as mean ± standard deviation for normally distributed data or median with interquartile ranges (IQR) for non-normally distributed data. Distribution normality was assessed using the Student’s *t* test. Non-normally distributed data was assessed using Mann-Whitney U test. Chi-square test or Fisher exact test was used for categorical variables. A two-tailed *P* < 0.05 was considered statistically significant.

## Results

### Patient Characteristics and Laboratory Data

From 22 January 2020 to 12 March 2020, a total of 328 CAP patients were admitted to the isolation ward during this period, of which 25 were confirmed COVID-19 patients and 169 (mean age 40.26 ± 14.42 years; 95 male) were enrolled in this retrospective study. Seventy of them were tested for the emergent viruses. The positive rates of EBV, HSV, and CMV were 23.43% (15/64), 22.06% (15/68), and 12.50% (8/64), respectively. A total of 6 patients were positive for two emergent viruses, 2 patients were positive for three emergent viruses. The demographic data and clinical features of these patients were listed in Table 1. The most common presenting symptoms of CAP patients were fever (98, 57.99%) and dry cough (55, 32.54%). However, there was no significant difference in clinical manifestations between the emergent viruses positive and negative groups (*P* > 0.05). The levels of albumin, hemoglobin, lymphocyte count, and percentage, CD4 + T lymphocyte count and percentage, CD4/CD8 ratio of the patients in emergent viruses positive group were lower than those of emergent viruses negative group (*P* < 0.05). The levels of PCT, CRP, IL-6, CD8 + T lymphocyte count, and percentage in emergent viruses positive group higher than those of emergent viruses negative group (*P* < 0.05). The most frequently underlying diseases of all these patients were hypertension (17, 10.06%) and diabetes (12, 7.10%). All patients with HIV infection have emergent viruses reactivation (see Table 2). For all CAP patients, there were four patients secondary to bloodstream infection and one patient secondary to abdominal infection, and the others might be caused by direct inhalation of pulmonary pathogenic organisms.

**TABLE 1:** Demographic and clinical characteristics in SARS-CoV-2-Negative community acquired pneumonia patients.

Variables	Total (*N* = 169)	Total (*N* = 70)	Emergent viruses positive (*N* = 28)	Emergent viruses negative (*N* = 42)	*P*-Value
Gender (M/F)	95/74	43/27	13/15	30/12	0.350
Age (year)	40.26 ± 14.42	44.33 ± 15.52	48.39 ± 15.98	41.62 ± 14.96	0.075
History of epidemiology (*n*, %)	77 (45.56)	7 (10.00)	0 (0.00)	7 (16.67)	**0.023**
Length of stay (d)	2 (1,5)	7.5 (6,13)	8.5 (6,19.75)	7 (6,9.25)	0.164
**Presenting symptoms and signs (*n*, %)**					
Fever	98 (57.99)	44 (62.86)	18 (64.29)	26 (61.90)	0.840
Dry cough	55 (32.54)	20 (28.57)	6 (21.43)	14 (33.33)	0.280
Fatigue	17 (10.06)	8 (11.43)	3 (10.71)	5 (11.90)	1.000
Expectoration	33 (19.53)	15 (21.43)	6 (21.43)	9 (21.43)	1.000
Pharyngalgia	30 (17.75)	6 (8.57)	0 (0.00)	6 (14.29)	0.074
Chest pain	8 (4.73)	3 (4.29)	1 (3.57)	2 (4.76)	1.000
Headache	11 (6.51)	7 (10.00)	5 (17.86)	2 (4.76)	0.107
Shortness of breath	18 (10.65)	12 (17.14)	6 (21.43)	6 (14.29)	0.437
Myalgia	8 (4.73)	3 (4.29)	1 (3.57)	2 (4.76)	1.000
**Laboratory data**					
WBC (× 10^9^/L)	8.47 (6.73,11.72)	8.34 (6.31,10.50)	8.21 (6.41,9.94)	8.34 (6.31,11.26)	0.154
*N* (%)	73.80 (63.60,80.40)	74.10 (65.50,83.70)	83.50 (65.80,88.10)	73.50 (65.50,81.89)	**0.001**
Albumin (g/l)	43.50 (39.60,46.05)	41.60 (34.00,44.90)	35.80 (28.80,41.10)	43.40 (40.00,46.20)	**0.000**
Hemoglobin (g/l)	141.00 (133.00,157.00)	140.00 (125.00,156.00)	124.00 (102.00,138.00)	148.00 (134.00,157.00)	**0.000**
Platelet (× 10^12^/L)	211.00 (168.00,264.00)	186.00 (141.00, 232.00)	186.00 (108.00,276.00)	182.00 (147.00,226.00)	0.515
Lymphocyte (%)	16.30 (10.60,26.50)	15.00 (8.90, 23.20)	10.80 (4.23,24.48)	16.60 (10.80,23.20)	**0.001**
Lymphocyte (× 10^9^/L)	1.33 (1.01,1.93)	1.17 (0.76,1.71)	0.88 (0.57,1.36)	1.36 (0.84,1.74)	**0.000**
Total bilirubin (μmol/l)	10.30 (7.50,14.00)	9.90 (7.30, 13.90)	9.50 (5.90,12.30)	10.50 (7.40,17.10)	**0.000**
eGFR (ml/min/1.73 m^2^)	103.70 (84.90,114.37)	96.38 (74.70, 111.62)	96.80 (71.40,114.90)	96.25 (82.80,110.11)	0.511
PCT (ng/ml)	0.06 (0.04,0.20)	0.08 (0.05,0.22)	0.14 (0.06,0.29)	0.06 (0.04,0.13)	**0.000**
CRP (mg/l)	30.80 (3.07,75.40)	29.30 (6.22,75.40)	47.50 (13.70,87.10)	24.00 (3.07,74.40)	**0.000**
IL-6 (pg/ml)	14.20 (2.31,31.70)	16.90 (2.32,31.7)	16.95 (2.14,30.80)	13.90 (5.28,40.80)	**0.000**
CD4 + T (%)	38.35 (33.80,46.00)	38.20 (31.9,44.80)	33.10 (22.80,46.90)	39.20 (35.00,44.80)	**0.000**
CD8 + T (%)	24.30 (19.10,29.70)	25.30 (19.90,30.30)	26.50 (22.50,41.30)	24.90 (19.00,28.15)	**0.000**
CD4/CD8 ratio (%)	1.67 (1.27,2.07)	1.60 (1.07,1.98)	1.55 (0.61,1.82)	1.63 (1.27,2.04)	**0.000**
CD4 + T count (cells/μl)	496.00 (322.00,725.00)	493.00 (327.00,661.00)	356.00 (126.00,493.00)	598.00 (452.00,753.00)	**0.000**
CD8 + T count (cells/μl)	324.00 (216.00,452.00)	299.00 (229.90, 438.00)	249.00 (170.00,496.00)	308.00 (273.00,438.00)	**0.001**

*WBC, white blood cell; N, neutrophil; PCT, procalcitonin; CRP, C-reactive protein; IL-6, Interleukin-6; eGFR: estimated glomerular filtration rate.*

*The items in bold are statistically significant.*

**TABLE 2:** The underlying diseases and risk factors of SARS-CoV-2-Negative community acquired pneumonia patients.

Underlying diseases and factors	Patients (*N* = 169) (*n*, %)	Total (*N* = 70) (*n*, %)	Emergent viruses positive (*N* = 28)	Emergent viruses negative (*N* = 42)	*P*-Value
Hypertension	17 (10.06)	9 (12.86)	4 (14.29)	5 (11.90)	1.000
Diabetes	12 (7.10)	10 (14.29)	6 (21.43)	4 (9.52)	0.183
Chronic kidney disease	9 (5.33)	7 (10.00)	4 (14.29)	3 (7.14)	0.426
Chronic lung disease	7 (4.14)	5 (7.14)	2 (7.14)	3 (7.14)	1.000
HIV infection	6 (3.55)	6 (8.57)	6 (21.43)	0 (0.00)	**0.003**
Cardiovascular disease	6 (3.55)	4 (5.71)	0 (0.00)	4 (9.52)	0.144
Malignancy	6 (3.55)	6 (8.57)	1 (3.57)	5 (11.90)	0.390
Chronic liver disease	6 (3.55)	2 (2.86)	1 (3.57)	1 (2.38)	1.000
Immunosuppressive drugs or corticosteroids	5 (2.96)	3 (4.29)	1 (3.57)	2 (4.76)	1.000
Hematology disease	3 (1.78)	2 (2.86)	2 (7.14)	0 (0.00)	0.157
Organ transplantation	3 (1.78)	2 (2.86)	1 (3.57)	1 (2.38)	1.000
Immune system disease	3 (1.78)	3 (4.29)	2 (7.14)	1 (2.38)	0.560

*The items in bold are statistically significant.*

### Pathogens Detection

The most common respiratory pathogens were *Mycoplasma pneumoniae* (12/163, 7.36%) and HRV (5/163, 3.07%). The positive rate for influenza was only 1.84%. For respiratory pathogens, there was no significant difference between the emergent viruses positive and negative groups (*P* > 0.05) (see Table 3).

**TABLE 3:** The detected pathogens of SARS-CoV-2-Negative community acquired pneumonia patients.

Pathogen detected	Total (*n*, %)	Total (*N* = 70) (*n*, %)	Emergent viruses positive (*N* = 28)	Emergent viruses negative (*N* = 42)	*P*-Value
Human rhinovirus (HRV)	5/163 (3.07)	2 (2.86)	1/27 (3.70)	1/40 (2.50)	1.000
Adenovirus (AdV)	0/163 (0)	0 (0)	0/27 (0)	0/40 (0)	–
Influenza A or B virus	3/163 (1.84)	0 (0)	0/27 (0)	0/40 (0)	–
Human metapneumovirus (hMPV)	3/163 (1.84)	0 (0)	0/27 (0)	0/40 (0)	–
Parainfluenza virus (PIV)	1/163 (0.61)	1 (1.43)	0/27 (0)	1/40 (2.50)	1.000
Respiratory syncytial virus (RSV)	0/163 (0)	0 (0)	0/27 (0)	0/40 (0)	–
Coronavirus	1/163 (0.61)	0 (0)	0/27 (0)	0/40 (0)	–
*Mycoplasma pneumoniae*	12/163 (7.36)	7 (10.00)	1/27 (3.70)	6/40 (15.00)	0.228
*Chlamydia pneumoniae*	4/163 (2.45)	1 (1.43)	0/27 (0)	1/40 (2.50)	1.000

### Findings of Initial Chest Computerized Tomography Scan

**FIGURE 2:**
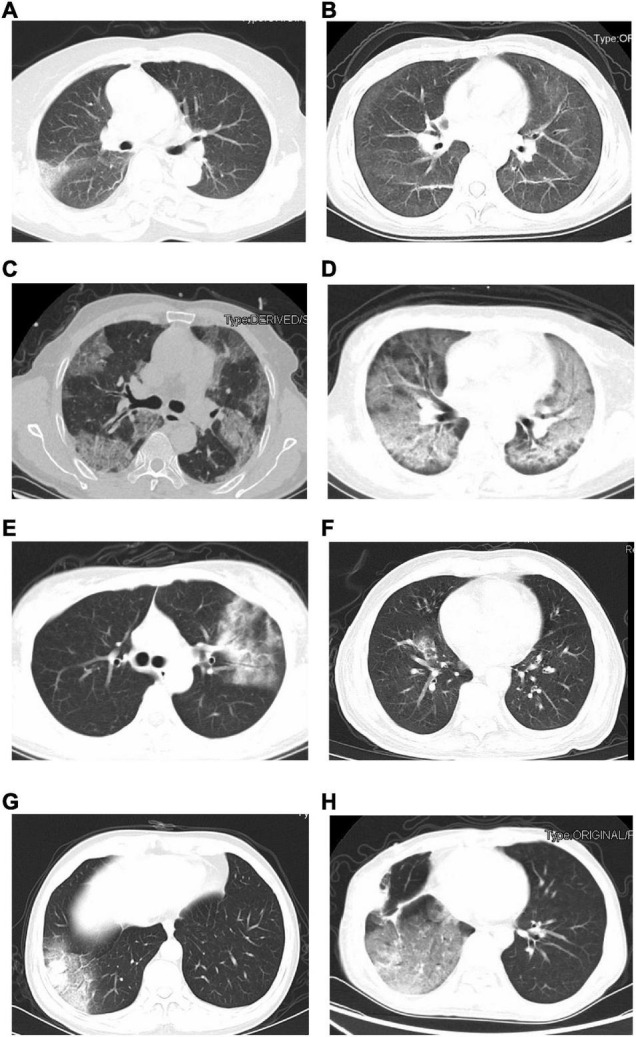
The initial chest CT features of SARS-CoV-2-Negative community acquired pneumonia patients (emergent viruses positive patients: **A–D** and emergent viruses negative patients: **E–H**). **(A)** Ground-glass opacities in right lung (bacterial pneumonia). **(B)** Ground-glass opacities, strip shadow, and partial interlobular septal thickening in double lung (AIDS). **(C)** Ground-glass opacities and nodules were widely distributed in both lungs, Crazy-Paving sign could be seen in some lesions (AIDS). **(D)** Diffuse distribution of patchy shadows, reticulation and multiple nodules in both lungs (systemic lupus erythematosus, lupus nephritis, and pneumocystis pneumoniae). **(E)** Ground-glass opacities, reticulation, and consolidation in left lungs (*Chlamydia pneumoniae* pneumonia). **(F)** Ground-glass opacities and consolidation in right lung (*Mycoplasma pneumonia*). **(G)** Ground-glass opacities and consolidation in right lung (alcoholic cirrhosis and bacterial pneumonia). **(H)** Chest CT scan shows Ground-glass opacities in right lungs (postoperative lung cancer, immune-associated pneumonia, and presumptive bacterial pneumonia).

The initial chest CT features of SARS-CoV-2-Negative CAP patients were diverse, as shown in Figure 2. Table 4 showed the detail of chest CT findings in the patients. The most common manifestations were GGO (91/169, 53.85%) and pulmonary nodules (88/169, 52.07%). The most frequently involved lobes were left lower lobe (106/169, 62.72%) and right lower lobe (98/169, 57.99%). More than half of the patients presented multifocal (130/169,76.92%), bilateral (95/169,56.21%) lung lesions. More lesions were inclined to distribute in the bilateral upper lobes and right middle lobe in the emergent viruses positive group than those in the emergent viruses negative group (*P* = 0.000).

**TABLE 4:** Initial chest CT findings of SARS-CoV-2-Negative community acquired pneumonia patients.

Morphology	Total (*N* = 169) (*n*, %)	Total (*N* = 70) (*n*, %)	Emergent viruses positive (*N* = 28)	Emergent viruses negative (*N* = 42)	*P*-Value
Ground-glass opacity	91 (53.85)	60 (85.71)	25 (89.29)	35 (83.33)	0.729
Nodule	88 (52.07)	41 (58.57)	17 (60.71)	24 (57.14)	0.766
Consolidation	31 (18.34)	18 (25.71)	6 (21.43)	12 (28.57)	0.503
Both of all	13 (7.69)	9 (12.86)	3 (10.71)	6 (14.29)	0.732
**With others**					
Patchy shadow	55 (32.54)	33 (47.14)	12 (42.86)	21 (50.00)	0.558
Interlobular septal thickening	8 (4.73)	6 (8.57)	2 (7.14)	4 (9.52)	1.000
Vascular enlargement	7 (4.14)	4 (5.71)	1 (3.57)	3 (7.14)	0.645
Air bronchogram	5 (2.96)	4 (5.71)	1 (3.57)	3 (7.14)	0.645
Fibrosis	4 (2.37)	2 (2.86)	1 (3.57)	1 (2.38)	1.000
Reticulation	9 (5.33)	6 (8.57)	4 (14.29)	2 (4.76)	0.209
Pleural thickening	14 (8.28)	7 (10.00)	5 (17.86)	2 (4.76)	0.107
Hydrothorax	12 (7.19)	10 (14.29)	7 (25.00)	3 (7.14)	0.077
Lymph node enlargement	18 (10.65)	15 (21.43)	8 (28.57)	7 (16.67)	0.234
**Distribution**					
Periphery distribution	14 (8.28)	8 (11.43)	5 (17.86)	3 (7.14)	0.252
Bilateral involvement	95 (56.21)	47 (67.14)	22 (78.57)	25 (59.52)	0.096
Multifocal involvement	130 (76.92)	54 (77.14)	24 (85.71)	30 (71.43)	0.246
Unifocal involvement	39 (23.08)	16 (22.86)	4 (14.29)	12 (28.57)	
**Lobe of lesion distribution**					
Left upper lobe	92 (54.44)	43 (61.43)	23 (82.14)	21 (50.00)	**0.006**
Left lower lobe	106 (62.72)	55 (78.57)	22 (78.57)	33 (78.57)	1.000
Right upper lobe	81 (47.93)	36 (51.43)	22 (78.57)	14 (33.33)	**0.000**
Right middle lobe	92 (54.44)	37 (52.86)	20 (71.43)	17 (40.48)	**0.011**
Right lower lobe	98 (57.99)	48 (68.57)	22 (78.57)	26 (61.90)	0.141
Bilateral upper lobes	64 (37.87)	33 (47.14)	21 (75.00)	12 (28.57)	**0.000**
Bilateral lower lobes	75 (44.38)	40 (57.14)	20 (71.43)	20 (47.62)	**0.049**

*The items in bold are statistically significant.*

### The Antimicrobial Treatment Strategies and Clinical Outcome

A total of 136 patients (136/169, 80.47%) received antimicrobial treatment empirically based on the community acquired pneumonia. Of the 136 patients, 104 patients (104/136, 76.47%) received monotherapy. Fluoroquinolone (80/136, 58.82%) and β-lactam monotherapy (20/136, 14.71%) were the most common antimicrobial regimens. More patients were received combination therapy in emergent viruses positive group (23/28, 82.14%) than those in emergent viruses negative group (2/42, 4.76%) (*P* < 0.05). More emergent viruses positive patients received fluoroquinolone monotherapy and fluoroquinolone combined with antiviral therapy (*P* < 0.05). Emergent viruses negative patients had a better clinical outcome (*P* < 0.05), as shown in Table 5.

**TABLE 5:** The antimicrobial treatment strategies and clinical outcome of SARS-CoV-2-Negative community acquired pneumonia patients.

Variables	Total (*n* = 169) (*n*, %)	Total (*N* = 70) (*n*, %)	Emergent viruses positive (*N* = 28)	Emergent viruses negative (*N* = 42)	*P*-Value
**Therapeutic regimen**					
No treatment	33 (19.53)	3 (4.29)	0 (0)	3 (7.14)	
Monotherapy	104 (61.54)	42 (60.00)	5 (17.86)	37 (88.10)	**0.000**
Combination therapy	32 (18.93)	25 (35.71)	23 (82.14)	2 (4.76)	
**Monotherapy**					
Fluoroquinolone monotherapy	80 (47.34)	31 (44.29)	3 (10.71)	28 (66.67)	**0.000**
β-lactam monotherapy	20 (11.83)	10 (14.29)	2 (7.14)	8 (19.05)	0.296
Macrolide monotherapy	1 (0.59)	1 (1.43)	0 (0)	1 (2.38)	1.000
Antiviral therapy	3 (1.78)	0 (0)	0 (0)	0 (0)	–
**Combination therapy**					
Fluoroquinolone + antiviral therapy	9 (53.25)	6 (8.57)	6 (21.43)	0 (0)	**0.003**
β-lactam + antiviral therapy	5 (2.96)	3 (4.29)	3 (10.71)	0 (0)	0.060
Fluoroquinolone + antifungal therapy	4 (2.37)	4 (5.71)	2 (7.14)	2 (4.76)	1.000
β-lactam + antifungal therapy	4 (2.37)	3 (4.29)	3 (10.71)	0 (0)	0.060
Fluoroquinolone + antiviral therapy + antifungal therapy	2 (1.18)	1 (1.43)	1 (3.57)	0 (0)	0.391
β-lactam + antiviral therapy + antifungal therapy	3 (1.78)	3 (4.29)	3 (10.71)	0 (0)	0.060
Combined sulfas	5 (2.96)	5 (7.14)	5 (17.86)	0 (0)	**0.008**
**Clinical outcome**					
Improvement or cure	164 (97.04)	66 (94.29)	24 (85.71)	42 (100.00)	**0.022**
Death or disease progression	5 (2.96)	4 (5.71)	4 (14.29)	0 (0)	

*The items in bold are statistically significant.*

## Discussion

Twenty years into the 21st century, COVID-19 is another outbreak of coronavirus in human beings after the outbreak of severe acute respiratory syndrome (SARS) in 2003 and the outbreak of Middle East Respiratory Syndrome (MERS) in 2012. Respiratory viruses are ubiquitous pathogens and can cause acute respiratory illnesses in all age groups, especially in young children and elderly adults (Falsey, 2007).

During the COVID-19 epidemic, many patients with respiratory symptoms and CT features of pneumonia were admitted to our hospital for further screening for COVID-19. Among SARS-CoV-2-Negative CAP patients, the detection rate of emergent viruses was higher than that of other pathogens. Although we had no direct evidence that lung lesions in these patients were associated with herpesviridae, they had herpesviridae infection or reactivation. Previous studies have shown that EBV is the most common reactivated virus in sepsis patients, with a plasma positive rate of 32–48% (Ong et al., 2017; Mallet et al., 2019). Reactivation of an endogenous latent virus (HSV, CMV, and EBV) was the most common of the opportunistic viral pathogens in critically ill patients and/or immunosuppressed patients (Cantan et al., 2019). In response to severe infection, individuals with previously normal immune function may develop a varying degrees of functional immunosuppression, triggering reactivation of the viruses (Brenner et al., 2012; Ong et al., 2017; Davila et al., 2018). At present, the exact significance and mechanism of viral reactivation remains to be determined. A recent study showed that EBV reactivation might be associated with the Sepsis Response Signature endotype (SRS1) immunocompromised sepsis transcriptomic endotype in patients with sepsis due to CAP (Goh et al., 2020). Emergent viruses reactivation may be related to ICU mortality, increased incidence of fungal infections, SOFA score and extended ICU stay (Walton et al., 2014; Mallet et al., 2019).

The most common presenting symptoms of these patients were fever and dry cough, which are similar to the symptoms of confirmed COVID-19 patients and other CAP patients (Prina et al., 2015; Guan et al., 2020). Therefore, it is difficult to distinguish the types of pneumonia from the clinical manifestations, and etiological diagnosis is more important for the diagnosis of pneumonia types (Ruuskanen et al., 2011). On admission, emergent viruses positive patients had lower CD4 + T lymphocyte count, albumin and hemoglobin values than those in the emergent viruses negative patients. Immunosuppression may have led to latent viruses reactivation, and infection-induced inflammation, immune dysfunction, and reactivated viruses may exacerbate the organ damage.

The most frequently underlying diseases of the CAP patients were hypertension and diabetes. which are similar to results of confirmed COVID-19 patients and CAP patients (Rivero-Calle et al., 2016; Wan et al., 2020; Yang J. et al., 2020). These groups are targeted for vaccination against influenza and pneumococcal disease (World Health Organization, 2008, 2012). The positive rates of respiratory viruses such as HRV, AdV, influenza virus, RSV were low in this study. The detection rate for influenza was only 1.84%, which was lower than that of previous years (Yang S. et al., 2020). An observational study also found influenza transmission declined substantially during COVID-19 outbreak in Hong Kong, which may be associated with non-pharmaceutical interventions including isolation, social distancing, and changes in population behavior (Cowling et al., 2020). Measures for preventing SARS-CoV-2 has positive effects on preventiong influenza virus infections. Atypical pathogens, especially *Mycoplasma pneumoniae*, are one of the main pathogens of CAP. *Mycoplasma pneumoniae* and *chlamydia pneumoniae* accounted for 9.82% in this study, which was different from other studies. Different countries, different periods and different populations may lead to different etiology of CAP (Yu and Fei, 2016).

The initial chest CT features were diverse in these CAP patients, and the most common manifestations were GGOs and pulmonary nodules in bilateral multiple lungs. Which were similar to the CT imaging features of patients with COVID-19 (Caruso et al., 2020; Xu et al., 2020). The imaging features of virus infections are usually multifocal GGOs. GGO is usually the CT manifestation of pathological diffuse alveolar damage (Chong et al., 2010). A nodular pattern of pneumonia may be seen in various infections (Reittner et al., 2003). Therefore, many patients in our study had pulmonary nodules. This study also found more emergent viruses positive patients have bilateral upper lobes involvement than emergent viruses negative patients. Patients with emergent virus reactivation have low cellular immune function, are more likely to progress to critical disease, and chest imaging lesions are more likely to involve multiple lung lobes. A recent study also showed the level of decreased CD4 + T cell may prompt the severity of CT imaging in patients with COVID-19 (Yang Y. et al., 2020). Due to the physiological anatomy of the lungs, the lesions of most CAP patients tend to occur in the lower lungs, and the upper lungs are less involved. Therefore, the upper lungs of patients with emergent virus reactivation are relatively more frequently involved.

A total of 33 patients did not receive treatment. These patients had no underlying disease, the diagnosis of viral pneumonia was considered clinically (excluding COVID-19 and influenza). Many viral pneumonia is a mild and self-limiting illness, and there is also no clear role for use of antivirals in treating viral CAP apart from influenza and HSV (Ruuskanen et al., 2011), so they were not given treatment. Most of the CAP patients received antimicrobial therapy and fluoroquinolone was the most common antimicrobial regimen. To date, there is no clear consensus on whether patients with obvious viral CAP need to be treated with antibiotics. Some experts recommend that all patients with pneumonia should be treated with antibiotics because it is impossible to rule out bacterial infections (Ruuskanen et al., 2011). Atypical pathogens are one of the main pathogens in CAP, and the incidence rate has increased. Though still controversial, empirical antibiotic coverage of atypical pathogens is recommended. Due to the high drug resistance rate of macrolide, fluoroquinolones such as moxifloxacin or levofloxacin should be considered in China (Yu and Fei, 2016). In addition, studies have shown that respiratory fluoroquinolones not only have potential antiviral activity against CMV, HSV, varicella-zoster virus, and SARS CoV-2, but also have immunomodulatory effects (Karampela and Dalamaga, 2020; Marciniec et al., 2020). Therefore, fluoroquinolones may be used in viral pneumonia. In terms of combination therapy, the pathogens of CAP were diverse, and CAP patients in the emergent viruses positive group had lower cellular immune function and were prone to fungal infection, so more patients in the emergent virus positive group were treated with combination therapy (Mandell, 2015; Cao et al., 2018; Upchurch et al., 2018; Metlay and Waterer, 2020; Aliberti et al., 2021). This study also found that the clinical outcomes of patients with herpesviridae reactivation were worse than those without herpesviridae reactivation. Therefore, if pneumonia develops in herpesviridae positive patients, early and aggressive treatment is needed to improve clinical outcomes.

This study have some limitations. First, it was a retrospective study and only part of patients had pathogen detection results. Only a small number of patients underwent fungal assessment and bacterial culture because of the allocation of medical resources at that time. Second, due to epidemic management, many out-of-town patients with CAP cannot come to our hospital for examination, which may lead to selection bias.

## Conclusion

Herpesviridae (HSV, CMV, and EBV) reactivation was common in SARS-CoV-2-Negative CAP patients. Emergent viruses positive patients have poorer cellular immune function, more severer conditions and poorer prognosis. Fluoroquinolones was the most commonly used antibiotic regimen for these patients. Pneumonia patients with emergent viruses reactivation need more aggressive treatment.

## Data Availability Statement

The original contributions presented in the study are included in the article/supplementary material, further inquiries can be directed to the corresponding author.

## Ethics Statement

The studies involving human participants were reviewed and approved by Ethics Committee of West China Hospital, Sichuan University. Written informed consent for participation was not required for this study in accordance with the national legislation and the institutional requirements.

## Author Contributions

XL and JQ conceived and designed the study. FH and HL participated in acquisition and analysis of data. JQ drafted the manuscript. XL and FH revised the manuscript critically for important intellectual content. All authors saw and approved the final version.

## Conflict of Interest

The authors declare that the research was conducted in the absence of any commercial or financial relationships that could be construed as a potential conflict of interest.

## Publisher’s Note

All claims expressed in this article are solely those of the authors and do not necessarily represent those of their affiliated organizations, or those of the publisher, the editors and the reviewers. Any product that may be evaluated in this article, or claim that may be made by its manufacturer, is not guaranteed or endorsed by the publisher.
